# Bempedoic Acid, the First-in-Class Oral ATP Citrate Lyase Inhibitor with Hypocholesterolemic Activity: Clinical Pharmacology and Drug–Drug Interactions

**DOI:** 10.3390/pharmaceutics16111371

**Published:** 2024-10-26

**Authors:** Nicola Ferri, Elisa Colombo, Alberto Corsini

**Affiliations:** 1 Department of Medicine, University of Padova, 35100 Padua, Italy; 2 Veneto Institute of Molecular Medicine, 35129 Padua, Italy; 3 Centro di Ricerca Coordinata sulle Interazioni Farmacologiche, 20122 Milan, Italy; elisa.colombo1@unimi.it (E.C.); alberto.corsini@unimi.it (A.C.); 4 Department of Pharmacological and Biomolecular Sciences “Rodolfo Paoletti”, University of Milan, 20122 Milan, Italy

**Keywords:** drug–drug interactions, bempedoic acid, statins, cytochrome P450, P-gp, organic anion transporter 2, organic anion transporter 3, organic anion-transporting polypeptide 1B1, organic anion-transporting polypeptide 1B3

## Abstract

Bempedoic acid is a new drug that improves the control of cholesterol levels, either as monotherapy or in combination with existing lipid-lowering therapies, and shows clinical efficacy in cardiovascular disease patients. Thus, patients with comorbidities and under multiple therapies may be eligible for bempedoic acid, thus facing the potential problem of drug–drug interactions (DDIs). Bempedoic acid is a prodrug administered orally at a fixed daily dose of 180 mg. The dicarboxylic acid is enzymatically activated by conjugation with coenzyme A (CoA) to form the pharmacologically active thioester (bempedoic acid–CoA). This process is catalyzed by very-long-chain acyl-CoA synthetase 1 (ACSVL1), expressed almost exclusively at the hepatic level. Bempedoic acid–CoA is a potent and selective inhibitor of ATP citrate lyase (ACL), a key enzyme in the biosynthetic pathway of cholesterol and fatty acids. The drug reduces low-density lipoprotein–cholesterol (LDL-C) (20–25%), non-high-density lipoprotein–cholesterol (HDL-C) (19%), apolipoprotein B (apoB) (15%), and total cholesterol (16%) in patients with hypercholesterolemia or mixed dyslipidemia. The drug has a favorable pharmacokinetics profile. Bempedoic acid and its metabolites are not substrates or inhibitors/inducers of cytochrome P450 (CYP450) involved in drug metabolism. On the other hand, bempedoic acid–glucuronide is a substrate for organic anion transporter 3 (OAT3). Bempedoic acid and its glucuronide are weak inhibitors of the OAT2, OAT3, and organic anion-transporting polypeptide 1B1 (OATP1B1) and 1B3 (OATP1B3). Thus, bempedoic acid could inhibit (perpetrator) the hepatic uptake of OATP1B1/3 substrate drugs and the renal elimination of OAT2 and OAT3 substrates and could suffer (victim) the effect of OAT3 transporter inhibitors, reducing its renal elimination. Based on these pharmacological characteristics, here, we describe the potential DDIs of bempedoic acid with concomitant medications and the possible clinical implications.

## 1. Introduction

Lipid-lowering drugs represent the first line of intervention for primary and secondary prevention of atherosclerotic cardiovascular disease (ASCVD). A Cholesterol Treatment Trialist (CTT) meta-analysis from randomized clinical trials firmly established that lowering low-density lipoprotein–cholesterol (LDL-C) with standard statin regimens reduced the 5-year relative incidence of major coronary events, coronary revascularizations, and ischemic strokes by about 20% for each mmol/L reduction in LDL-C, and that additional reductions, obtained with more intensive statin regimens, further reduced the incidence of these major vascular events [1]. In addition, the clinical benefit of LDL-C reduction with statin therapy was confirmed in individuals who had a 5-year risk lower than 10%, even in those who had no previous history of vascular disease, diabetes, or chronic kidney disease [2].

The combination of statins with either ezetimibe, which reduces the intestinal uptake of dietary and biliary cholesterol by inhibiting the Niemann–Pick C1-like protein 1 (NPC1L1) or monoclonal antibodies anti-proprotein convertase subtilisin/kexin type 9 (PCSK9), further reduced the LDL-C levels and major cardiovascular events [3,4,5]. Thus, the clinical data demonstrated that, independently from the pharmacological approach, the more aggressive lowering of LDL-C is associated with increased benefits in reducing atherosclerotic disease burden, supporting the notion of ‘the lower, the better’ [6,7]. Indeed, in patients with ASCVD, the long-term achievement of lower LDL-C levels with monoclonal anti-PCSK9 evolocumab, down to <20 mg/dL (<0.5 mmol/L), has been associated with a lower risk of cardiovascular outcomes with no significant safety concerns [8]. The most recently approved hypocholesterolemic drug is represented by the small molecule bempedoic acid [9]. Bempedoic acid has shown effective hypocholesterolemic activity, either as monotherapy or in combination with existing lipid-lowering therapy, in a broad spectrum of patients at high cardiovascular risk [10,11,12]. This drug has been demonstrated to significantly reduce the major cardiovascular events in statin-intolerant patients, either in primary or secondary prevention [13,14]. Here, we described the pharmacological properties of bempedoic acid and the potential drug–drug interactions that might occur in patients with multiple comorbidities.

## 2. Mechanism of Action of Bempedoic Acid

**Figure 1:**
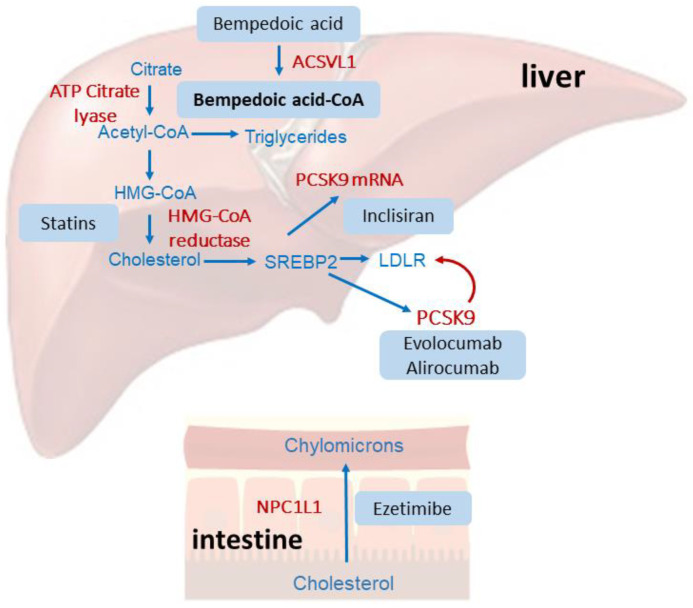
Schematic representation of the mechanism of action of bempedoic acid compared to statins, ezetimibe, and anti-PCSK9 therapies. Statins, by inhibiting the hydroxy-methyl-glutaryl–CoA (HMG-CoA) reductase, reduce cholesterol biosynthesis in the liver, determining the activation of the transcription factor SREBP2, which drives the expression of the LDL receptor. Statins also induce the expression of PCSK9, which can degrade the LDL receptor and thus partially reduce the hypocholesterolemic effect of statins. Monoclonal antibodies (evolocumab and alirocumab) bind and inhibit PCSK9, while inclisiran reduces its synthesis, interfering with its mRNA. Bempedoic acid acts by inhibiting the ACLY, and thus reduces cholesterol biosynthesis, which activates a similar cellular response to that observed with statins. Finally, ezetimibe interacts with the intestinal cholesterol transporter NPC1L1, reducing its absorption from the diet.

Bempedoic acid is a prodrug that requires enzymatic activation by the very-long-chain acyl–CoA synthetase 1 (ACSVL1, encoded by the SLC27A2 gene) enzyme, almost exclusively expressed in the liver (Figure 1). Thus, dicarboxylic acid is conjugated with coenzyme A (CoA) to form the pharmacologically active thioester (bempedoic acid–CoA) in the liver where it acts as a potent and selective inhibitor of ATP citrate lyase (ACLY), a key enzyme in the biosynthetic pathway of cholesterol and fatty acids [9]. Thus, bempedoic acid and statins act on two different enzymatic steps in the same metabolic pathway. For this reason, the lipid-powering effect of bempedoic acid appears to be more pronounced in statin-intolerant patients [10]. Concentration–response studies have shown that bempedoic acid–CoA inhibits cholesterol synthesis in cultured hepatocytes, with a IC_50_ value equal to 10 µM [9]. In response to cholesterol biosynthesis, hepatocyte activates the transcription factor sterol regulatory element-binding protein 2 (SREBP2), which drives the expression of LDL receptors and PCSK9. Thus, the upregulation of LDL receptors increases the LDL catabolism with a concomitant reduction in plasma LDL cholesterol levels.

## 3. Pharmacokinetic Properties

### 3.1. Absorption

The administration of multiple doses of bempedoic acid (180 mg/day) resulted in C_max_ and AUC values of between 24.8 ± 6.9 μg/mL and 348 ± 120 μg·h/mL, respectively (Table 1) [15]. After 7 days, at a steady state, no time-dependent changes were observed in the pharmacokinetic profile of the drug [16]. Bempedoic acid is a weak acid with high solubility at intestinal pHs, resulting in rapid oral absorption from the gastrointestinal tract. The pH-dependent solubility characteristics and high membrane permeability are clinically manifested in rapid oral absorption, with a median time-to-peak concentration of 3.5 h [16,17,18,19]. The bioavailability of bempedoic acid is 95%, and it is not influenced by the presence of food (Table 1) [20].

### 3.2. Distribution

Bempedoic acid has a volume of distribution of 18 L and a plasma protein binding of 99% (Table 1) [15,16].

### 3.3. Metabolism

Bempedoic acid is reversibly converted to the active metabolite ETC15228 (AUC of the metabolite is 18% compared to bempedoic acid). ETC15228, a keto metabolite of bempedoic acid, is converted into the active molecule thioester CoA conjugate by the same mechanism of activation of bempedoic acid [21]. Metabolism of ETC15228 by ACSVL1 leads to the formation of ETC15228–CoA, a selective inhibitor, in the liver of the ACLY, with a potency similar to bempedoic acid–CoA. Although ETC15228 thioester and bempedoic acid–CoA are equipotent, ETC15228 plasma exposure is approximately 72% lower than bempedoic acid levels with a mean steady state C_max_ of 2.8 ± 0.9 ng/mL compared to a C_max_ of 20.6 ± 6.1 μg/mL [20]. The corresponding time–concentration profile of ETC15228 indicates a T_max_ of 11.0 h with an elimination half-life (t_1/2_) of 31.1 h, significantly slower than the t_1/2_ of 19–21 h of bempedoic acid (Table 1) [15].

**Figure 2:**
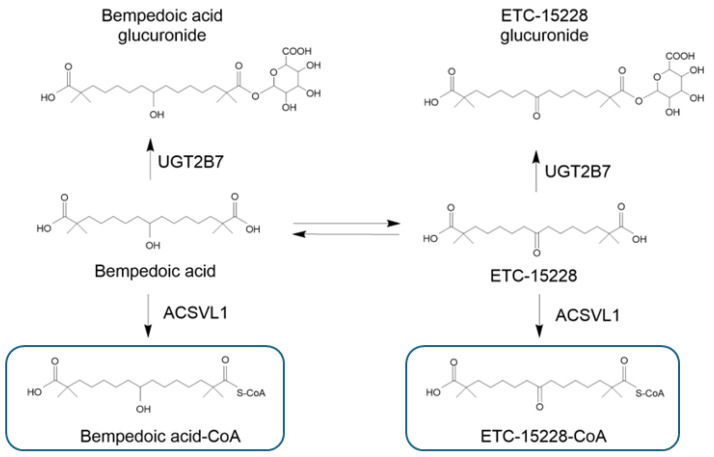
Main metabolites of bempedoic acid. Bempedoic acid and its keto metabolite ECT-15228 are both substrates of the UGT2B7 enzyme, which forms the two glucuronide derivatives. The enzyme ACSVL1 also converted the bempedoic acid and ECT-15228 into their active metabolites. UGT-2B7: UDP-Glucuronosyltransferase-2B7; ACSVL1: very-long-chain acyl–CoA synthetase 1. Bempedoic acid–CoA and ETC-15228–CoA are the active metabolites formed selectively in the liver.

Bempedoic acid and its metabolites are not substrates or inhibitors/inducers of cytochrome P450 (CYP450) involved in drug metabolism but are metabolized through conjugation with glucuronic acid by UDP-Glucuronosyltransferase-2B7 (UGT2B7) (Figure 2) [15,20]. Based on AUC_inf_ exposure, the metabolites bempedoic acid–glucuronide, ETC15228, and ETC15228–glucuronide represent approximately 28.9%, 12.1%, and 10.4%, respectively, of the total plasma radioactivity detectable after administration of 180 mg of [^14^C]–bempedoic acid [20].

### 3.4. Elimination

At steady state, after once-daily administration, the bempedoic acid total clearance is equal to 11.2 mL/min. Renal clearance of the parental drug represents less than 2% of the total clearance. However, between 70% and 30% of the total dose is found in the urine and feces, respectively, although bempedoic acid represents less than 5% of the administered dose (feces and urine). The half-life of bempedoic acid at a steady state is 19–21 h [20].

After administration of [^14^C]–bempedoic acid, the majority of the radioactivity is found in urine; thus, renal clearance is the most representative route of elimination for the drug. The most abundant analyte in urine is bempedoic acid–glucuronide (Figure 2), which represents approximately 37.3% of the total radioactivity excreted in 120 h [20]. The metabolite ETC15228–glucuronide and bempedoic acid are also excreted in urine, and represent from 10% to 14% and from 0.9% to 3.3% of the total radioactivity, respectively [20].

The clearance of bempedoic acid was studied in patients with mild, moderate, and severe renal impairment [18]. Although exposure (AUC) to bempedoic acid was increased approximately two-fold in subjects with renal impairment, compared to subjects with normal renal function, no safety problems with the drug were observed. This is due to the fact that, despite an increase in AUC in subjects with renal insufficiency, C_max_ values did not change compared to subjects with normal renal function. A possible explanation relies in the fact that, in conditions of renal insufficiency, bempedoic acid is mostly eliminated via the bile in the form of glucuronide, with subsequent enterohepatic recirculation and lengthening of the plasma half-life after a reduction in clearance. No dosage adjustment of bempedoic acid is suggested in patients with mild or moderate renal insufficiency [15].

## 4. General Considerations on DDI with Bempedoic Acid

DDIs can be related to the pharmacokinetics or pharmacodynamics of a drug. Taking into consideration the pharmacodynamics of bempedoic acid, it is conceivable to hypothesize a pharmacological interaction with drugs or phytotherapies that act on the same ACLY enzyme or that inhibit its activation from prodrug to active drug by ACSVL1.

Considering the pharmacokinetic profile, bempedoic acid is neither a substrate nor an inhibitor of P-glycoprotein [16]; therefore, no interactions with inducers or inhibitors of this transporter are expected. Furthermore, bempedoic acid is a weak acid with high solubility at intestinal pH; for this reason, interactions with proton pump inhibitors (PPIs) or other antacids are not expected.

Bempedoic acid, as well as its active metabolite and glucuronide form, are not substrates of commonly characterized drug transporters, with the exception of bempedoic acid–glucuronide, which is an OAT3 substrate [22]. However, bempedoic acid and its glucuronide are weak inhibitors of OATP1B1/3, while only bempedoic acid is an inhibitor of the OAT2 and OAT3 transporters [15]. These characteristics indicate that bempedoic acid could inhibit (perpetrator) the liver uptake of OATP1B1/3 substrate drugs and the renal elimination of OAT2/3 substrate drugs. Furthermore, bempedoic acid itself could suffer (victim) the effect of OAT3 inhibitors, reducing its renal clearance. Probenecid, an inhibitor of UGT and OAT3 transporters, has been studied to evaluate the potential effect of OAT3 inhibitors on the pharmacokinetics of bempedoic acid. Administration of 180 mg of bempedoic acid with steady-state probenecid resulted in a 1.7-fold increase in bempedoic acid exposure and a 1.9-fold increase in bempedoic acid active metabolite exposure [17]. These elevations are not considered to be clinically meaningful and do not impact dosing recommendations [15,17]. Indeed, human pharmacokinetics of bempedoic acid are linear over the dose range of 120–240 mg, and doses up to 240 mg/day (1.3 times the approved recommended dose) have been administered in clinical trials, with no evidence of dose-limiting toxicity [15,20]. In addition, a single dose of bempedoic acid was also well-tolerated in subjects with severe renal impairment that experienced a 2.2-fold increase in drug exposure [18]. Thus, relevant interactions between bempedoic acid and other OAT3 inhibitors are not expected to be clinically relevant.

Finally, by inhibiting the OAT2/3 renal transporter, bempedoic acid determines a minor and reversible increase in uric acid and creatinine plasma levels, effects that can be added to those related to other drugs. The minor increase incidence of cholelithiasis in patients treated with bempedoic acid, observed exclusively in the phase 3 clinical trial CLEAR outcomes [13], could deteriorate by the co-treatment with other drugs with similar side effects [13], such as the peroxisome proliferator-activated receptor α (PPARα) agonist fenofibrate [23,24].

## 5. Pharmacodynamic DDIs with Bempedoic Acid

As previously described, bempedoic acid acts by inhibiting the ACLY and, thus, cholesterol biosynthesis. Possible DDIs may derive from drugs or chemical entities that act on the same enzyme and thus interfere with bempedoic acid. In addition, molecules that inhibit or are a substrate of ACSVL1 may interfere with bempedoic acid activation and, thus, its activity. For instance, ACLY is inhibited by hydroxycitric acid present in *Garcinia cambogia*, a natural product utilized to reduce bodyweight and plasma cholesterol levels [25]. Thus, the addition of this nutraceutical product may potentially interfere with the activity of bempedoic acid and should be avoided. Additionally, omega 3 fatty acids are substrates of ACSVL1 [9], the hepatic enzyme required for the activation of bempedoic acid. It is, therefore, reasonable to predict a possible interaction between bempedoic acid and omega 3, including the most recently approved highly purified eicosapentaenoic acid ethyl ester, icosapent ethyl [26].

## 6. Pharmacokinetics DDIs with Bempedoic Acid

To report a most comprehensive revision of the DDIs with bempedoic acid, we screened for therapies that have either an inhibitor/inducer activity on drug transporter OAT3 or that are a substrate for OATP1B1/B3 and OAT2/3.

### 6.1. Potential DDIs with Lipid-Lowering Drugs

To reach the appropriate LDL-C level, the current guidelines indicate the use of combination therapies with different lipid-lowering drugs, including statins, ezetimibe, PCSK9 inhibitors, fenofibrate, and bempedoic acid [27]. In addition, lifestyle changes should be pursued to control plasma lipid levels. From these recommendations, it is important to rule out or take into consideration the potential DDIs with functional foods or hypocholesterolemic drugs with bempedoic acid.

Considering the inhibitory activity of bempedoic acid on liver transporters OATP1B1/B3, drugs that are a substrate for these two transporters may undergo significantly higher exposure, with a possible increase in their side effects. Among many classes of drugs, it is important to remember that all statins are substrates of OATP1B1 and OATP1B3 and are susceptible of single nucleotide polymorphisms (SNPs) determining a partial loss-of-function phenotype [28], an effect mainly observed with simvastatin [29]. The pharmacokinetic interactions between bempedoic acid 180 mg and simvastatin 40 mg, atorvastatin 80 mg, pravastatin 80 mg, and rosuvastatin 40 mg were evaluated in clinical trials [15]. Administration of a single 40 mg dose of simvastatin with 180 mg of steady-state bempedoic acid resulted in a two-fold increase in simvastatin acid exposure. Elevations from 1.4-fold to 1.5-fold in AUC of atorvastatin, pravastatin, and rosuvastatin (administered as single doses) and/or their major metabolites were observed when co-administered with bempedoic acid (Table 2). From this evidence, the simvastatin dose should be limited to 20 mg daily (or 40 mg daily for patients with severe hypercholesterolemia and who are at a high risk of cardiovascular complications) when combined with bempedoic acid. In contrast, the interactions with atorvastatin, rosuvastatin, and pravastatin were not considered to be clinically relevant, and thus their combination with bempedoic acid is possible. In addition, both lovastatin and its analog monacolin K present in the red yeast rice preparations are predicted to not interact with bempedoic acid.

### 6.2. Potential DDIs with Immune-Modulating and Antineoplastic Agents

It is important to point out that bempedoic acid and its glucuronide derivative are inhibitors but not substrates of OATP1B1/1B3; therefore, a variation in their systemic exposures is not expected with inhibitors of these transporters (Table 3). Thus, bempedoic acid, differently from statins, can be administered with cyclosporine, which is an inhibitor of CYP3A4, P-gp, and OATP1B1/1B3 [36,37,38]. Similarly, everolimus and tacrolimus are inhibitors of OATP1B1 and should not alter the pharmacokinetics of bempedoic acid [39,40].

The immunomodulators leflunomide and teriflunomide are both inhibitors of OAT3 [41,42], being the first that also affect OATP1B1/B3 transporters [41]. For this double action, teriflunomide increased systemic exposure of rosuvastatin by 2.5-fold, and it is indicated to reduce the dose of statin by 50% when co-administered with this immunomodulator [41]. The inhibition of renal OAT3 could also increase the systemic exposure of bempedoic acid–glucuronide, while the plasma levels of uric acid could potentially be controlled by leflunomide, whose treatment is associated with its higher fractional excretion [43].

Mycophenolate mofetil, another immunomodulator, is a substrate for OAT1, OAT3, MRP2, and BCRP [44], and its co-administration could determine a significant drug interaction with bempedoic acid by increasing its systemic exposure. This combination could also be associated with a higher incidence of hyperuricemia and gout (Table 3) [44,45].

Darolutamide, an androgen receptor inhibitor, is an inhibitor of OATP1B1/B3; indeed, the co-administration of rosuvastatin should be avoided. Similar interactions can be expected with other statins, and bempedoic acid could be a valid therapeutic alternative in these patients [46]. Enzalutamide is metabolized by CYP2C8 and CYP3A4 and can induce different enzymes and drug transporters, such as CYP3A4, CYP2B6, CYP2C9, CYP2C19, UGT1A1, MRP2, OATP1B1, and P-gp (Table 3) [47]. For this reason, it is expected to interact with statins metabolized by CYP3A4 (atorvastatin and simvastatin), and the induction of OATP1B1 could potentiate their hepatic uptake, while bempedoic acid should not be affected by these changes.

Tucatinib, the HER2 inhibitor, can induce several enzymes (CYP2B6, CYP3A4, CYP2C9, CYP2C19, or UGT1A1) and inhibits the drug transporters MRP2, OCT1, and OAT3 (Table 3) [48]. Thus, tucatinib and bempedoic acid are both inhibitors, but not substrates of OAT3, and their pharmacokinetics should not be altered when administered simultaneously.

**Table 3:** Potential DDIs of bempedoic acid with immunosuppressant and antineoplastic agents.

Drug	Effect on CYP450 and Drug Transporters	Effect on Bempedoic Acid	Effect on Interacting Drug	Expert Opinion
Immunosuppressant drugs				
Cyclosporine	Inhibitor of CYP3A4 and P-gp, OATP1B1/1B3 [36,37,38].	No significant effect predicted.	No significant effect predicted.	Bempedoic acid inhibits OATP1B1/1B3, but it is not a substrate of these transporters. Thus, no interaction is predicted with OATP1B1/1B3 inhibitors or inducers.
Tacrolimus	Metabolized by CYP3A4 and substrate of P-gp [36,37,38].	No significant effect predicted.	No significant effect predicted.	No drug interaction.
Everolimus, sirolimus	Everolimus is a substrate of CYP3A4 and weak inhibitor of P-gp and CYP2D6 [49]. Sirolimus is metabolized by CYP3A4 and is a substrate of P-gp [50]. Both are inhibitors of OATP1B1 [39,40].	No significant effect predicted.	No significant effect predicted.	Bempedoic acid inhibits OATP1B1/1B3, but it is not a substrate of these transporters. Thus, no interaction is predicted with OATP1B1/1B3 inhibitors or inducers.
Leflunomide	Metabolized by CYP1A2, CYP2C19, and CYP3A4. Inhibitor of OAT3 [42].	Leflunomide could increase the exposure of bempedoic acid.	No significant effect predicted.	It is recommended to have caution when administered with an OAT3 substrate like bempedoic acid–glucuronide.
Teriflunomide	Inhibitor of CYP2C8. Inducer of CYP1A2. Inhibitor of OAT3, OATP1B1/B3 [41].	Teriflunomide could increase the exposure of bempedoic acid.	No significant effect predicted.	The inhibition of OAT3 could increase the AUC of bempedoic acid–glucuronide and potentially the plasma levels of uremic acid and creatinine.
Mycophenolate mofetil	Metabolized by UGT1A9. Substrate of OAT1, OAT3, MRP2, and BCRP [44].	No significant effect predicted.	Bempedoic acid could increase the exposure of mycophenolate mofetil.	Bempedoic acid could reduce the renal excretion of mycophenolate mofetil by competing with OAT1 and OAT3. Both drugs increase uric acid plasma levels. Higher incidence of gout is predicted.
Anticancer drugs				
Tamoxifen	Metabolized by CYP3A4. CYP2D6 metabolism leads to the formation of the active molecule endoxifen [51]. Inhibitor of P-gp and is a substrate of OATP1A2 [52].	No significant effect predicted.	No significant effect predicted.	No drug interaction.
Taxanes (paclitaxel, docetaxel)	Paclitaxel is a substrate of CYP2C8 and CYP3A4. Docetaxel is a substrate of CYP3A4. Both are substrates of OATP1B1/1B3.	No significant effect predicted.	Bempedoic acid could increase the exposure of docetaxel and paclitaxel.	Bempedoic acid inhibits OATP1B1/1B3 and may increase the exposure of docetaxel and paclitaxel.
Darolutamide	Metabolized by CYP3A4 and UGT1A9. Substrate of P-gp and BCRP. Inhibitor of OATP1B1/B3 [46].	No significant effect predicted.	No significant effect predicted.	Co-administration of rosuvastatin with darolutamide should be avoided unless there is no alternative therapy. Consider drugs which are not substrates for OATP1B1 and OATP1B3. Bempedoic acid inhibits OATP1B1/1B3, but it is not a substrate of these transporters. Thus, no interaction is predicted with OATP1B1/1B3 inhibitors or inducers.
Imatinib, crizotinib and tucatinib	Inhibitor of CYP3A4 and P-gp. Imatinib inhibits CYP2D6, UGT2B17, and, partially, UGT1A1 [53]. Tucatinib could induce CYP2B6, CYP3A4, CYP2C9, CYP2C19, or UGT1A1, and inhibits MRP2, OCT1, and OAT3 [48].	Tucatinib could increase the exposure of bempedoic acid.	No significant effect predicted.	The inhibition of OAT3 could increase the AUC of bempedoic acid–glucuronide and potentially the plasma levels of uremic acid and creatinine.
Enzalutamide	Metabolized by CYP2C8 and CYP3A4. Inducer of CYP3A4, CYP2B6, CYP2C9, CYP2C19, UGT1A1, MRP2, OATP1B1, and P-gp [47]. Possible inhibition of MRP2, OCT1, and OAT3.	Enzalutamide could increase the exposure of bempedoic acid.	No significant effect predicted.	Induction of OATP1B1 is not expected to potentiate the hepatic uptake and activity of bempedoic acid. Enzalutamide is an OAT3 inhibitor. This could increase the AUC of bempedoic acid–glucuronide and potentially the plasma levels of uremic acid and creatinine.

### 6.3. Potential DDIs with Antiviral, Antibiotic, and Antifungal Agents

The combination lopinavir/ritonavir strongly inhibits CYP3A4 and the hepatic transporters OATP1B1/B3 (Table 4) [54,55]. These effects may determine a clinically relevant interaction with statins, but not with bempedoic acid, which could be considered a valid alternative for HIV patients with hypercholesterolemia. Asunaprevir, glecaprevir, grazoprevir, and voxilaprevir are anti-HCV drugs and substrates for OATP1B1 and OATP1B3. Thus, bempedoic acid is expected to increase their exposure and side effects [15]. The antiretroviral drug dolutegravir is another strong inhibitor of the OAT3 transporter, and its co-administration could prolong the exposure of bempedoic acid (Table 4) [56].

A single dose of rifampicin has been shown to inhibit the drug transporters OATP1B1/B3, while multiple doses determine a strong induction of CYP3A4, CYP2C19, P-gp, and UGT [57]. All of these effects are not expected to have an impact on the pharmacokinetics of bempedoic acid. Similar conclusions can be drawn for erythromycin and clarithromycin, strong inhibitors of CYP3A4, P-gp, and OATP1B1/B3 [58,59,60].

**Table 4:** Potential DDIs of bempedoic acid with antiviral, antibiotic, and antifungal agents.

Drug	Effect on CYP450 and Drug Transporters	Effect on Bempedoic Acid	Effect on Interacting Drug	Expert Opinion
Antivirals, antibiotics, antifungals				
Dolutegravir/lamivudine/abacavir	No inhibition of CYP450. Dolutegravir inhibits OAT1 and OAT3.	Dolutegravir may increase plasma concentrations of drugs excreted through OAT3, such as bempedoic acid.	No significant effect predicted.	The inhibition of OAT3 could increase the AUC of bempedoic acid–glucuronide and potentially the plasma levels of uremic acid and creatinine.
Atazanavir and ritonavir	Potent inhibitors of CYP3A4. Inhibitors of glucuronidation [61,62].	Possible reduction in glucuronide metabolite of bempedoic acid.	No significant effect predicted.	Inhibition of glucuronidation could prolong the half-life of bempedoic acid.
Lopinavir and ritonavir	Potent inhibitors of CYP3A4. Inhibitors of OATP1B1 and OATP1B3 [54,55].	No significant effect predicted.	No significant effect predicted.	Bempedoic acid inhibits OATP1B1/1B3, but it is not a substrate of these transporters. Thus, no interaction is predicted with OATP1B1/1B3 inhibitors or inducers.
Asunaprevir, glecaprevir, grazoprevir and voxilaprevir	Substrate of OATP1B1 and OATP1B3	No significant effect predicted.	Possible increase in their exposure.	Bempedoic acid, by inhibiting OATP1B1/1B3, may increase the exposure of anti-HCV drugs.
Antibiotic				
Rifampicin	Single dose inhibits OATP1B1 and OATP1B3. Strong inducer of CYP3A4, CYP2C19, P-gp and UGT [57].	No significant effect predicted.	No significant effect predicted.	Bempedoic acid inhibits OATP1B1/1B3, but it is not a substrate of these transporters. Thus, no interaction is predicted with OATP1B1/1B3 inhibitors or inducers. The induction of UGT could increase the metabolism of bempedoic acid.
Erythromycin and clarithromycin	Inhibitors of CYP3A4 and P-gp. Inhibitors of OATP1B1 and OATP1B3 [58,59,60,63].	No significant effect predicted.	No significant effect predicted.	Bempedoic acid inhibits OATP1B1/1B3, but it is not a substrate of these transporters. Thus, no interaction is predicted with OATP1B1/1B3 inhibitors or inducers.
Antifungal				
Ketoconazole, itraconazole and voriconazole	Potent inhibitors of CYP3A4, P-gp, and BCRP. Ketoconazole is a potent inhibitor of OATP1B1, OATP1B3, OAT3, OCT1, and OCT2, and a weak inhibitor of OAT [64].	No significant effect predicted.	No significant effect predicted.	An increase in systemic exposure of bempedoic acid–glucuronide may be expected by OAT3 inhibition of ketoconazole. Itraconazole and voriconazole do not appear to inhibit this transporter [65,66].

### 6.4. Potential DDIs with Antidiabetic Agents

The sodium glucose co-transporter 2 (SGLT2) inhibitor empagliflozin is a substrate of the OAT3, OATP1B1, and OATP1B3 transporters; thus, its elimination can be inhibited by bempedoic acid, an OAT3 inhibitor (Table 5). Nevertheless, this interaction is not predicted to be relevant, since probenecid, a potent OAT3 inhibitor, and gemfibrozil, another potent inhibitor of OAT3 and OATP1B1/1B3, did not show a significant interaction with empagliflozin [67]. Others SGLT2 inhibitors (ertugliflozin, dapagliflozin, and canagliflozin) are not substrates of OAT3, thus excluding any potential interaction with bempedoic acid (Table 5). Considering other antidiabetic drugs, among the dipeptidyl peptidase 4 (DDP4) inhibitors, sitagliptin is a substrate of both P-gp and OAT3 [68]. Bempedoic acid, by inhibiting OAT3, could increase the systemic exposure of sitagliptin. No interaction is expected with other DDP4 inhibitors (Table 5).

Phase 1 clinical trials have not found significant pharmacokinetic interaction between metformin and bempedoic acid [15,19]. This could have been predicted by taking into consideration the fact that metformin is metabolized by UGT1A1, UGT1A3, and by CYP3A4 and it is substrate for MRP2 and BCRP [69]. In addition, no changes were observed on the glycemic control of metformin when administered with bempedoic acid. In this regard, it is important to point out that metformin is an indirect activator of the AMP-dependent kinase (AMPK) by changing the ratio of ATP/AMP [70], while bempedoic acid–CoA has been suggested to directly activate this metabolic pathway [9].

**Table 5:** Possible DDIs of bempedoic acid with antidiabetic agents.

Drug	Effect on CYP450 and Drug Transporters	Effect on Bempedoic Acid	Effect on Interacting Drug	Expert Opinion
Antidiabetic drugs				
Metformin	Metabolized by UGT1A1 and partially by UGT1A3 and CYP3A4. Substrate of MATE-1, MATE-2K, and OCT2 [69].	No significant effect predicted.	No significant effect predicted.	No pharmacokinetic and pharmacodynamic interaction observed between metformin and bempedoic acid [15,19].
Glyburide	Substrate of CYP2C9. Substrate and inhibitor of OATP1B1/1B3.	No significant effect predicted.	Possible increase in glyburide exposure.	Bempedoic acid inhibits OATP1B1/1B3, but it is not a substrate of these transporters. Thus, no significant interaction is predicted.
Repaglinide	Substrate of CYP2C8 and OATP1B1/1B3.	No significant effect predicted.	Possible increase in repaglinide exposure.	Bempedoic acid inhibits OATP1B1/1B3, but it is not a substrate of these transporters. Thus, no significant interaction is predicted.
SGLT2 (ertugliflozin, dapagliflozin, empagliflozin, canagliflozin)	Metabolized by UGT. Empagliflozin is a substrate of OAT3 and OATP1B1/1B3 [67,71]. The dapagliflozin metabolite is a substrate of OAT3 [72].	No significant effect predicted.	No significant effect predicted.	Although empagliflozin is a substrate for OAT3, OATP1B1, and OATP1B3, no significant interaction is predicted with bempedoic acid. Indeed, the strong OAT3 inhibitor probenecid showed a minimal effect on the exposure of empagliflozin [67].
DPP4 inhibitors (sitagliptin, vildagliptin, saxagliptin, linagliptin, alogliptin)	Saxagliptin is metabolized by CYP3A4/5. Linagliptin is a weak inhibitor of CYP3A4 and substrate of P-gp [73]. Sitagliptin is a substrate of P-gp and OAT3 [68].	No significant effect predicted.	Possible increase in sitagliptin exposure.	Bempedoic acid, by inhibiting the OAT3, could reduce the clearance of sitagliptin. No interactions are predicted with other DDP4 inhibitors.
GLP1 agonists (liraglutide, exenatide, semaglutide)	Proteolytic metabolism.	No significant effect predicted.	No significant effect predicted.	No drug interaction.

### 6.5. Potential DDIs of Bempedoic Acid with Cardiovascular Drugs

Warfarin has several drawbacks, such as a delayed onset of anticoagulant action, a narrow therapeutic index, and an unpredictable and variable response related to single nucleotide polymorphisms in CYP2C9 and VKORC1. Due to the narrow therapeutic index, warfarin is extremely susceptible to DDIs with inhibitors and inducers of CYP2C9. Although bempedoic acid should not alter the exposure of warfarin, their combination deserves a close monitoring of INR (Table 6). Differently, no DDIs are predicted in bempedoic acid and direct oral anticoagulants (DOAC), whose pharmacokinetics are mainly determined by the expression and function of P-gp [74].

Safe co-administration can also be predicted with antiarrhythmic agents, while the systemic exposure of bosentan, a drug approved for the treatment of pulmonary arterial hypertension, can be increased by bempedoic acid, since it is a substrate of OATP1B1 and OATP1B3 (Table 6) [15].

### 6.6. Potential DDIs of Bempedoic Acid with Antidepressant, Antipsychotic, and Antiepileptic Drugs

Different classes of antidepressant and antipsychotic drugs are extensively metabolized by CYP450 enzymes and may inhibit CYP2D6; however, both selective serotonin reuptake inhibitors (SSRI) and tricyclic antidepressants (TCA) are not influenced by the inhibition of OATP1B1/B3 and OAT3 transporters and, therefore, can be administered with bempedoic acid. Two antipsychotic drugs, clozapine and olanzapine, are associated with dyslipidemias, and, thus, the use of bempedoic acid can be envisioned to control plasma lipid levels (Table 6). Antiepileptic drugs, including carbamazepine, phenobarbital, and phenytoin, are considered to be strong inducers of CYP450 and P-gp and can interact with statins [76], while no changes in the pharmacokinetics of bempedoic acid are expected (Table 7). Finally, St. John’s Wort is a strong inducer of different cytochromes and P-gp, while its active constituent, hyperforin, has been found to inhibit OATP1B1/B3. Thus, this phytotherapy is contraindicated with statins, but can be considered in the presence of bempedoic acid.

### 6.7. Bempedoic Acid and Uric Acid: Potential DDI

Considering bempedoic acid’s side effects, its administration in combination with loop and thiazide diuretics may determine a further increase in uric acid levels and, potentially, cases of gout [79]. Other drugs that can increase the uric acid levels and incidences of gout are reported in Table 8.

## 7. Conclusions

Bempedoic acid represents a novel and effective hypocholesterolemic agent to be prescribed either as monotherapy or in combination with existing lipid-lowering therapies in a broad spectrum of patients at high cardiovascular risk. The drug has shown an excellent safety profile with selective activation in hepatocytes, avoiding side effects on skeletal muscles and glycemic homeostasis. Its use is associated with increased plasma levels of creatinine and uric acid due to the mild and reversible inhibition of the renal transporters OAT2 and OAT3. The drug has a favorable pharmacokinetics profile whose metabolism and disposition do not rely on CYP450 and the P-gp transporter, although it is a weak inhibitor of hepatic transporters OATP1B1 and OATP1B3. Thus, its use could be safer than statins, considering their muscle-related side effects and their CYP450-mediated metabolism. Although direct clinical evidence of DDIs with bempedoic acid is limited to statins [15], metformin [19], and probenecid [17], the results of a 2-year follow-up of a real-world evaluation of the effectiveness and safety of bempedoic acid reported a good safety profile for the drug. Overall, 0.3% (5/973) of patients had serious ADRs considered to be related to bempedoic acid. Among these adverse drug reactions, myalgia was the most common, occurring in 3.5% (34/973) of patients [92]. Thus, the safety profile of bempedoic acid in this real-world population is consistent with that observed in the CLEAR clinical trial program [11,13,93], suggesting a minor incidence of clinically relevant drug interactions in complicated and polytreated patients.

Bempedoic acid–glucuronide is also a substrate of OAT3, and thus any drugs with inhibitory actions on this transporter might reduce its clearance and potentially increase the plasma levels of uremic acid and creatinine. Given its favorable profile, bempedoic acid can be easily associated with other therapies and potentially be utilized in substitutions with statins in cases of intolerance.

## Figures and Tables

**Table 1:** Pharmacokinetic characteristics of bempedoic acid.

Target	ATP-citrate lyase (ACLY)
Prodrug	Yes, active metabolite is bempedoic acid–CoA produced in the liver by the very-long-chain acyl–CoA synthetase 1 (ACSVL1)
IC_50_	10 µmol/L
Bioavailability	95%
Effect of food	Not influenced
Vd	18 L
Proteins bound	99%
C_max_	24.8 ± 6.9 μg/mL
AUC	348 ± 120 μg·h/mL
T_max_	3.5 h
Half-life time	19–21 h
Metabolism	UGT2B7 no CYP450
Substrate/inhibitor P-gp	No
Substrate of other transporters	OAT3
Inhibitor of other transporters	OAT2, OAT3, OATP1B1, OATP1B3
Renal excretion	70%
Posology	180 mg OD

**Table 2:** Potential DDIs of bempedoic acid with lipid-lowering agents.

Drug	Effect on CYP450 and Drug Transporters	Effect on Bempedoic Acid	Effect on Interacting Drug	Expert Opinion
Hypocholesterolemic drugs				
Statins (pravastatin, simvastatin, atorvastatin, rosuvastatin)	All substrate of OATP1B1 [30].	No significant effect predicted.	A 2-fold increase in AUC simvastatin 40 mg. A 1.4-fold increase in AUC atorvastatina 80 mg. A 1.4-fold increase in AUC pravastatin 80 mg. A 1.5-fold increase of AUC rosuvastatina 40 mg [15].	Do not exceed 40 mg of simvastatin with bempedoic acid. Use rosuvastatin, atorvastatin, or other statins [15].
Fibrates (fenofibrate, gemfibrozil)	Gemfibrozil inhibits UGT2B7 [31,32,33].	Possible reduction in glucuronide metabolite of bempedoic acid.	No significant effect predicted.	Avoid gemfibrozil. Fenofibrate causes cholelithiasis. Possible increased risk of cholelithiasis with bempedoic acid [13].
mAbs anti-PCSK9 (evolocumab, alirocumab)	No effect on CYP450 and drug transporters [34].	No significant effect predicted.	Bempedoic acid increases PCSK9 expression and potentially the clearance of evolocumab and alirocumab [34].	This interaction is not considered clinically relevant.
siRNA anti-PCSK9 (inclisiran)	No effect on CYP450 and drug transporters [35].	No significant effect predicted.	No significant effect predicted.	No drug interaction.
Icosapent ethyl (omega 3)	Icosapent ethyl or EPA could compete with bempedoic acid for ASCVL1 [9].	Icosapent ethyl could interfere with the activation of bempedoic acid by ASCVL1.	No significant effect predicted.	Possible reduction in the hypocholesterolemic effect of bempedoic acid in the presence of Icosapent ethyl.

**Table 6:** Possible DDIs of bempedoic acid and cardiovascular drugs.

Drug	Effect on CYP450 and Drug Transporters	Effect on Bempedoic Acid	Effect on Interacting Drug	Expert Opinion
Anticoagulant drugs				
Warfarin	Metabolized by CYP3A4, 2C9, 2C19, and 1A2. Not a substrate for P-gp.	No significant effect predicted.	No significant effect predicted.	Monitor INR in patients taking warfarin and bempedoic acid.
DOAC (dabigatran, apixaban, rivaroxaban, edoxaban)	All are substrates of P-gp. Apixaban and rivaroxaban are metabolized by CYP3A4 and are substrates of BCRP and ABCG2 [75].	No significant effect predicted.	No significant effect predicted.	No drug interaction.
Antihypertensive drugs				
Bosentan	Substrate of OATP1B1 and OATP1B3 [15].	No significant effect predicted.	Possible increase in bosentan exposure.	Bempedoic acid inhibits OATP1B1 and OATP1B3, increasing the exposure of bosentan.
Antiarrhythmic drugs				
Amiodarone	Weak inhibitor of P-gp and moderate inhibitor of CYP3A4.	No significant effect predicted.	No significant effect predicted.	No drug interaction.
Dronedarone	Inhibitor of P-gp e CYP3A4.	No significant effect predicted.	No significant effect predicted.	No drug interaction.
Digoxin	Substrate of P-gp.	No significant effect predicted.	No significant effect predicted.	No drug interaction.
Diltiazem, verapamil,	Inhibitor of P-gp and moderate inhibitor of CYP3A4.	No significant effect predicted.	No significant effect predicted.	No drug interaction.

**Table 7:** Possible DDIs of bempedoic acid with antidepressant, antipsychotic, and antiepileptic agents.

Drug	Effect on CYP450 and Drug Transporters	Effect on Bempedoic Acid	Effect on Interacting Drug	Expert Opinion
Antidepressive drugs				
SSRI (fluoxetine, paroxetine, sertraline, venlafaxine, citalopram, escitalopram)	Fluoxetine, paroxetine, and sertraline inhibit CYP2D6.	No significant effect predicted.	No significant effect predicted.	No drug interaction.
TCA (amitriptyline, imipramine, clomipramine, amoxapine, desipramine, nortriptyline)	Metabolized by CYP3A4, CYP2C9, and CYP2D6.	No significant effect predicted.	No significant effect predicted.	No drug interaction.
Antipsychotic drugs				
Haloperidol, clozapine, perphenazine, risperidone, quetiapine, chlorpromazine	Metabolized by CYP3A4 and 2D6. Clozapine also by CYP2C19.	No significant effect predicted.	No significant effect predicted.	No drug interaction. Clozapine and olanzapine cause dyslipidemias: possible treatment with bempedoic acid.
Antiepileptic drugs				
Carbamazepine, phenobarbital, phenytoin, levetiracetam	Potent inducer of CYP3A4 and P-gp. Levetiracetam has a minor effect.	No significant effect predicted.	No significant effect predicted.	No drug interaction.
Phytotherapies				
St John’s Wort	Potent inducer of CYP3A4 and P-gp. Iperforin inhibits P-gp, OATP1B1, and OATP1B3 [77,78].	Uncertain effect.	No significant effect predicted.	Co-administration is possible.

**Table 8:** Drugs that can increase the uric acid levels and incidences of gout in the presence of bempedoic acid.

Drug	Mechanism
Aspirin (low dose)	At low doses (60–300 mg), aspirin reduces uric acid excretion and can induce hyperuricemia, while higher doses are uricosuric [80]. At low doses, aspirin acts as an exchange substrate and facilitates urate reabsorption, whereas at high doses it acts as an inhibitor of urate reabsorption [81]. Aspirin interacts with MRP4 [82], OAT1, and OAT3 [83].
Ticagrelor	Ticagrelor and its metabolite are weak inhibitors of the urate transporter URAT1 and the OAT3 transporter. The metabolite also inhibits OAT1 [84].
Cancer chemotherapy	Massive cytotoxic effect of tumor cells, with consequent release of urate [83].
Diuretics	Loop and thiazide diuretics inhibit OAT1- and OAT3-mediated uric acid secretion [85]. Furosemide and hydrochlorothiazide are substrates of the uric acid transporter MRP4 [82]. Reduction in plasma volume [83]. Hydrochlorothiazide increases uric acid reuptake via the OAT4 transporter [86].
Cyclosporine	Increased reabsorption of uric acid in the proximal tubule via the OAT1 transporter [87,88]. Decreased glomerular filtration rate secondary to vasoconstriction of afferent arterioles [89].
Tacrolimus	Reduce acid uric excretion [90].
Mycophenolate mofetil	Increased uric acid due to the inhibition of IMPDH (Inosine-5′-monophosphate dehydrogenase) [45].
Testosterone	Increases uric acid reabsorption through induction of Smct1 (SLC5A8) [91].
